# When and Which Dark Traits Shine? A Multidimensional Review of the Dark Triad in Organizational Research

**DOI:** 10.1002/pchj.70130

**Published:** 2026-11-13

**Authors:** Sajida Hafeez, Faisal Qadeer

**Affiliations:** ^1^ Faisalabad Business School National Textile University Faisalabad Pakistan; ^2^ Hailey College of Commerce The University of Punjab Lahore Pakistan

**Keywords:** dark triad, Machiavellianism, narcissism, positive outcomes, psychopathy, systematic review

## Abstract

The present research addresses the grand challenge entrenched in the upside of the dark triad (narcissism, Machiavellianism, and psychopathy). To date, reviews have rarely examined the positive aspects of the dark triad. Therefore, this systematic review identifies key mechanisms, contextual conditions, and theoretical frameworks that may leverage the dark triad constructively and mitigate its potentially detrimental manifestations. We summarize the available evidence on the positive side of the dark triad in the domain of organizational behavior using data from 107 studies representing 51,124 respondents. Finally, we review theories used in studying the dark triad and identify significant gaps and trends. We emphasize the necessity of reframing dark triad as potentially context‐dependent, thus expanding the scope of both theory and research methodologies. Reframing the dark triad literature offers an opportunity to advance the field by integrating a range of constructs, theories, and methodologies, which ultimately contributes to a more comprehensive understanding of the dark triad's context‐dependent association with work outcomes.

## 1 Introduction

Over the past two decades, dark triad literature has firmly established that dark triad traits are unavoidable in organizations. Therefore, labelling these traits as dark and assuming they are inherently harmful may lead scholars to design studies that confirm their pre‐existing beliefs. Moreover, contemporary personality philosophy contends that labelling personalities as inherently ‘good’ or ‘bad’ is an oversimplification (O'Reilly and Pfeffer 2021). However, a purely negative characterization may overlook variation in the organizational outcomes associated with these traits and the contextual conditions that shape DT expression. The term Dark Triad encompasses three complex personality traits, namely narcissism, Machiavellianism, and psychopathy. Narcissism is characterized by a strong sense of self‐importance, the need for extreme admiration, and a high sense of entitlement (Raskin and Hall 1979). Machiavellianism is characterized by manipulative, strategic, cynical, and highly self‐interested tendencies, particularly in an organizational setting (Christie and Geis 1970; LeBreton et al. 2018). Psychopaths exhibit traits of lack of empathy, lack of concern, impulsivity, absence of remorse (Hare 1980), manipulation, callousness, erratic lifestyle, and antisocial behavior (Jones and Paulhus 2014; LeBreton et al. 2018). The Dark Triad has predominantly been known for manipulation, lack of empathy, and self‐interest (Jones and Paulhus 2014). Emerging research has focused on examining the negative outcomes of Dark Triad such as malevolence (Muris et al. 2017) and instructed faking. Recently, Joshi et al. (2024) reviewed the Dark Triad literature, including from multiple perspectives in the student sample. Also, there remain very limited systematic reviews and meta‐analyses on the positive outcomes of the Dark Triad, such as happiness (Zheng and MacCann 2023), prosocial behaviors (Lin and Xie 2023) and well‐being (Blasco‐Belled et al. 2024). Smith et al. (2018) have made strides in addressing this gap by conducting a systematic review that elucidates the curvilinear effects of Dark Triad personality traits. However, Smith et al. (2018) reviewed only a few areas, such as leadership emergence, strategic management, human resources, and entrepreneurship. In the context of organizational behavior, only narcissists' adaptiveness, Mach's challenging OCB, and Mach's pro‐social behaviors were found as upsides of the dark personalities by Smith et al. (2018). To date, few systematic reviews or meta‐analyses have investigated the Dark Triad in depth, with positive outcomes in organizational behavior and organizational psychology. The current study aims to fill this gap by reviewing the Dark Triad field from multiple angles. This study seeks to provide a summary of the swiftly developing literature, employing a multidisciplinary approach to evaluate the current state of findings and potential future directions. Therefore, this research raises the following three questions:

1. What research has been conducted on the positive side of the Dark Triad, and how has it progressed the literature of Organizational Behavior?
2. What is the intellectual structure of the Dark Triad, its themes, theories, and perspectives, and what are its notable contributions?
3. Which specific topics, methodological approaches, and theoretical frameworks should be used in the future when investigating the upside of the Dark Triad of personality?

To this end, the study makes three contributions. First, the review adopts a comprehensive approach by initially examining all research on the Dark Triad to identify its potential aspects. Through this multidisciplinary synthesis, we aim to trace the evolution of the Dark Triad, highlight potential benefits across domains, and identify gaps in the literature and the study's contribution. Second, we systematically map the literature on the positive outcomes associated with the Dark Triad, particularly within the realm of organizational behavior. Third, we delve into the foundational theories that underpin this literature, aiming to guide future scholars in making meaningful contributions to this field. Lastly, we highlight specific constructs, theories, methods, and empirical approaches for scholars to consider in their research on the positive aspects of the Dark Triad. By meticulously mapping the current state of the literature, we aim to bridge established perspectives with promising avenues for future exploration and analysis.

Recent systematic reviews on Dark Triad personality traits (e.g., Borgholthaus et al. 2023; Bartolo and Powell 2024; Zheng and MacCann 2023) have provided useful insights, often emphasizing specific outcomes or perspectives. However, the literature is rather scattered, so we are unable to generalize about the contextual potential of the Dark Triad. In line with methodological guidance, drawing on a larger body of literature, ideally 40 or more relevant studies, can provide a stronger foundation for developing robust conclusions (Paul et al. 2021).

Fields that are more mature and established provide clearer insights regarding their respective research area. Moreover, a clarion call has arisen from multiple researchers across the domains and within the academic community, urging scholars to investigate the delineating circumstances, methods, predictors, situations, and factors that have the potential to ameliorate the negative impact of the Dark Triad (Schyns et al. 2023). This research seeks to address this call and contribute to a more nuanced understanding of the interaction between the Dark Triad and workplace dynamics.

## 2 The Dark Triad: A Constructive Standpoint

The term Dark Triad was coined in 2002 comprised narcissism, Machiavellianism, and psychopathy. These three traits are antagonistic yet theoretically distinct (Furnham et al. 2013). The extant body of literature is replete with investigations into the merits of positive traits and the detriments associated with the Dark Triad. However, contemporary perspectives within personality psychology increasingly contend that the classification of personalities into rigid categories of “good” and “bad” is an overly simplistic reduction (O'Reilly and Pfeffer 2021). Instead, it is assumed that all personalities inherently possess both positive and negative tendencies. In the wake of the initial conceptualization of the Dark Triad, research endeavors have taken a notable step forward by exploring the relationship between the Dark Triad and favorable outcomes (Djeriouat 2024). The characterization of a Narcissistic personality pertains to individuals who exhibit a distinct sense of entitlement and a grandiose perception of self. Certain theorists, such as Heinz Kohut (2009), have conceptualized narcissism as a defence mechanism geared toward self‐protection. Notably, from the broader second‐order construct of the Dark Triad, narcissism is the oldest part and has its origins in the ancient Greek legend of Narcissus, who fell in love with his image. The theoretical underpinnings of subclinical narcissism were derived and developed by Raskin and Hall in 1979. The prominent facets of superiority, self‐importance, grandiosity, admiration, and entitlement come from clinical disorders. Over time, these facets were refined meticulously based on extensive empirical research and culminated in a comprehensive inventory to assess narcissistic personality.

Researchers have acknowledged a shift in understanding the Dark Triad perspective (Morf and Rhodewalt 2001). Consequently, narcissists have been found to exhibit characteristics such as, self‐encouragement, intelligence, entitlement, dominance, and attractiveness (Muris et al. 2017). There has been a rapid increase in narcissism research, and articles focused on narcissists' dark personalities are growing and well‐documented in Western cultures (Germain and Germain 2018). Narcissism is referred to as a “contemporary epidemic” aimed toward social change that occurred in industrial and post‐industrial times. In organizational behavior, a few studies have shown the positive aspect of the Dark Triad. Narcissistic adaptiveness is recognized as a brighter aspect of narcissism that helps to manage uncertain and emergent conditions (Smith et al. 2018). In addition, narcissistic executives are not always bad for organizational health. This evolving research trajectory reflects a growing recognition of the need for a deeper understanding of the intricate dynamics at play within the Dark Triad personality construct and its implications for the organizational context. (Smith et al. 2018).

The conceptualization of the Machiavellian personality trait stems from Niccolò Machiavelli's renowned personality. This concept underwent a significant transformation through the efforts of Christie and Geis in 1970, as they adapted Machiavelli's ideas into the conventional framework of personality assessment, refining it and achieving consensus on its constituent items. The core characteristics that define Machiavellianism encompass a strong inclination towards forethought, strategic planning, and manipulative tendencies, and a goal‐directed effort defines the positive aspects of Machiavellianism as explained by Jones and Paulhus (2014).

In organizational behavior, Machiavellianism involves a complex interplay of behaviors. On the one hand, it is associated with challenging organizational citizenship behavior (OCB), while on the other hand, it manifests pro‐social behaviors (Becker and O'Hair 2007). Psychopathy represents the most nascent construct within the subclinical personality domain. Key characteristics of psychopathy generally encompass a penchant for thrill‐seeking, a marked lack of concern for others, and high levels of job involvement (Hirschfeld and Van Scotter 2019). While some research on secondary psychopathy highlights associations with neuroticism (Miller et al. 2010; Vize et al. 2018), neuroticism is not conventionally regarded as a core defining feature of psychopathy. Hare's (1985) self‐report psychopathy scale offers a valuable instrument, effectively distinguishing between clinical psychopathy and its subclinical counterparts. The overarching features that unite the Dark Triad personality traits include social tendencies, self‐confidence, and an inclination towards self‐promotion.

In previous research, the concept of the corporate psychopath has gained popularity. Corporate psychopathy has been found in high‐order positions as chief executive officers and top management (Sutton 2013). Corporate psychopaths get quick promotions to top‐level positions. The presence of corporate psychopaths is relatively higher in corporate samples, that is, 5.9% as compared to the community sample (1.2%) (Babiak et al. 2010). One distinguishing feature of subclinical psychopaths, which includes corporate psychopaths, is their exceptional capacity for comprehending and manipulating the behaviors and emotions of others. This keen understanding of human behavior confers a distinct advantage in the realms of business and politics, a phenomenon elucidated by Babiak et al. (2010).

However, within the field of organizational behavior, psychopathy has not yet been examined for its potential outcomes. The existing literature predominantly concentrates on the adverse ramifications of psychopathy, leaving a significant gap in our understanding of its potential benefits and advantages within the organizational context. Further exploration in this area may shed light on the nuanced interplay of psychopathy in organizational behavior, encompassing both its adverse and beneficial aspects.

The latest research primarily focuses on the potential positive aspects of the Dark Triad by identifying situations, conditions, and contexts in which the Dark Triad has proven to be strategic and beneficial (LeBreton et al. 2018). Despite being the least favorable trait of psychopathy, along with other traits, psychopathy has been found most often among employees and employers (Boddy 2015). Psychopaths are more likely to achieve thriving outcomes if they work in a coherent behavioral style. For psychopaths, the achievement of success is prioritized, even if it comes at the expense of others (DePaulo 2010). They possess compelling and charming personalities with high‐risk tendencies (DePaulo 2010).

## 3 Methodology

We adhered to the Preferred Reporting Items for Systematic Review and Meta‐Analysis Protocols (PRISMA‐P) guidelines by Moher et al. (2015) for conducting systematic reviews and meta‐analyses, as used in a recent review of the Dark Triad by Blasco‐Belled et al. (2024). These guidelines were used in the planning, execution, and reporting phases of the current review.

### 3.1 Search Strategy

At the beginning of the literature review, an exhaustive search was conducted to identify all potentially relevant studies from diverse sources, including databases, reference lists, and citations. Specifically, four prominent databases, namely JSTOR, Taylor & Francis, ScienceDirect, and Google Scholar, were used to retrieve pertinent articles. Researchers often choose platforms like Google Scholar, ScienceDirect, JSTOR, and Taylor & Francis because they offer a wide range of studies on topics like the Dark Triad in psychology and consistently yield richer outcomes compared to other platforms (Brownell et al. 2021; Moor and Anderson 2019). The initial search was executed in March 2022, followed by a subsequent search in November 2023 to refresh the systematic exploration of the literature. An additional search was conducted in March 2025 to identify more recent studies. The final search was conducted in July 2025. The search strategy used a comprehensive set of keywords and search strings, and the search process was very thorough to ensure all relevant studies were included. The detailed list of search terms used is tabulated in Appendix A of the Supporting Information. The Google Scholar search yielded enormous results and proved to be the most effective research engine, producing 12,840 articles on the search terms as depicted in Appendix A. The complete search strings are provided in the Supporting Information Appendix A. The search strings included 48 strings, with the number of search results for each term mentioned in the Supporting Information Appendix A. The search strings included terms such as “advantages of Dark Triad characteristics, advantages of embracing the Dark Triad, advantages of leveraging Dark Triad strategies, benefits from Dark Triad attributes, benefits of cultivating Dark Triad, benefits of Dark Triad, benefits of integrating Dark Triad tactics, beneficial attributes of the Dark Triad, bright outcomes from Dark Triad mind‐set, advantages of Machiavellianism leadership, benefits of narcissistic personality traits, bright outcomes of psychopathic decision‐making”.

### 3.2 Selection of Studies

Studies were included if they examined at least one Dark Triad trait (narcissism, Machiavellianism, or psychopathy), assessed a positive outcome, and were empirical studies published between 2011 and 2025. Studies were excluded if they did not examine the Dark Triad, focused exclusively on negative outcomes, lacked empirical data, were duplicates, or did not meet the specified publication or language criteria. The study selection process followed a systematic screening procedure, as illustrated in Figure 1. The initial database search identified 28,870 records from Google Scholar (*n* = 12,840), ScienceDirect (*n* = 9274), Taylor & Francis (*n* = 5527), and JSTOR (*n* = 1229). An additional 11 records were identified through other sources. After removing 17,820 duplicate records, 11,061 records remained for title and abstract screening. Of these, 10,843 records were excluded because they did not meet the predefined eligibility criteria, leaving 218 articles for full‐text retrieval. Of these, 13 articles could not be retrieved, while 205 full‐text articles were assessed for eligibility. At this stage, 98 articles were excluded because they lacked organizational context (*n* = 26), did not report positive outcomes associated with the Dark Triad (*n* = 18), or overlapped with or cross‐referenced other studies (*n* = 54). Consequently, 107 studies were included in the final systematic review.

**FIGURE 1:**
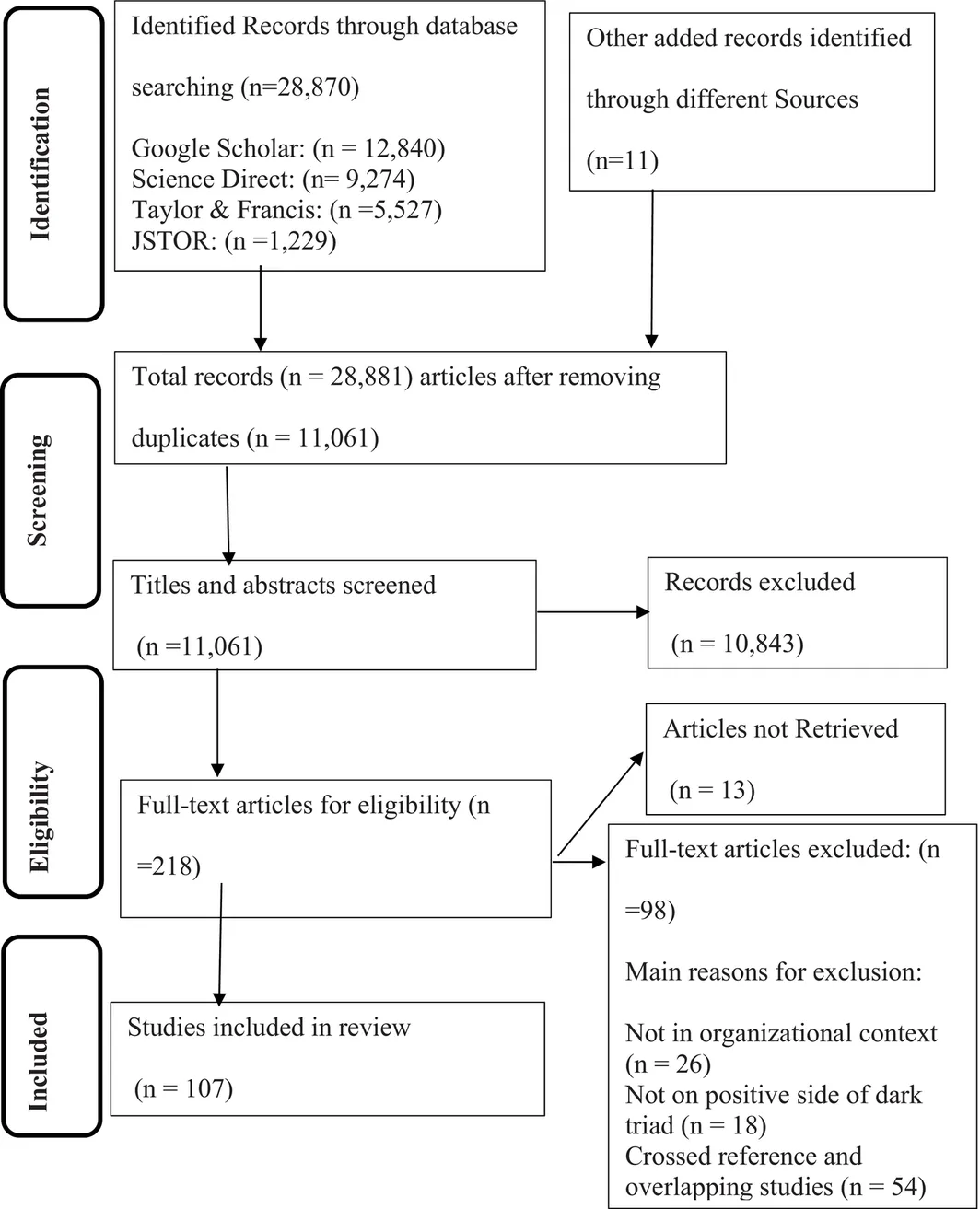
PRISMA diagram of literature search and selection process.

Initial screening and selection were conducted by the first author, and the second author independently reviewed and verified the findings for consistency and data validity. To avoid discrepancies, consensus was reached through discussion of the reviewed studies. The extracted information was subsequently organized thematically to identify common patterns, similarities, and contradictory findings across the reviewed studies. All relevant studies from other sources were incorporated into the review process and identified as pertinent research through additional means, such as reference lists or citations. In addition to exclusions based on failing to meet the minimum criteria, all studies that showed some degree of potential relevance following the preliminary study evaluation progressed to the eligibility assessment phase. Full texts of these studies were examined thoroughly to assess whether they met the defined inclusion criteria.

The 107 studies used in this final synthesis comprise both studies found through the original search and studies that were added through supplemental searches. Appendix B of the Supporting Information contains details of the 107 included studies. The study selection process is illustrated in Figure 1 to provide clarity and transparency regarding how the studies evaluated in this systematic review were either included or excluded at every step of the process.

## 4 Upsides of Dark Triad: A Brief Review

Personality psychology, organizational behavior, and management have been the primary outlets for the positive side of DT. Personality & Individual Differences Journal remains dominant, with a substantial share of the studies included in this review (e.g., Crysel et al. 2013; Jonason, Wee, and Li 2015; Smith and Webster 2018; Fernández‐del‐Río et al. 2020; Szabó et al. 2022; Bulbuc and Visu‐Petra 2024). However, contributions from management journals are increasing, including *Journal of Management* (Zagenczyk et al. 2014; Belschak et al. 2015), *Journal of Business Ethics* (Castille et al. 2018; Laurijssen et al. 2024), *Leadership Quarterly* (Rovelli and Curnis 2021), *Journal of Applied Psychology* (Wang et al. 2023), *Organizational Behavior and Human Decision Processes* (Carnevale et al. 2018; Wang et al. 2024), and *Human Resource Management Review* (Nassif 2019).

Secondly, several researchers have made recurring contributions, progressively developing this research stream. Peter Karl Jonason has been a prominent researcher investigating the motivational and adaptive characteristics of the Dark Triad, particularly regarding mating, risk‐taking, job satisfaction, creativity, and compassion (Jonason, Wee, and Li 2015; Jonason, Richardson, and Potter 2015). Similarly, Smith and Webster have made influential contributions, demonstrating that narcissism can promote OCB, career‐related outcomes, and innovation under supportive organizational conditions (Smith and Webster 2017, 2018; Webster and Smith 2019). Szabó further extended the literature by demonstrating positive associations among the Dark Triad, resilience, mental toughness, and organizational attitudes (Szabó et al. 2022, 2023). Collectively, the field has gradually shifted from documenting isolated positive outcomes toward developing a more systematic understanding of the conditions under which Dark Triad traits may become organizational assets.

The literature examines the Dark Triad from multiple perspectives, including individual‐level characteristics, associated outcomes, and factors that help explain the behavior of individuals with Dark Triad traits, as summarized in Appendix B of the Supporting Information. These examinations are categorized into self‐trait levels, emotional aspects, behavioral consequences, and, notably, creative outputs.

At the self‐trait level, self‐regulation stands out, with narcissistic individuals demonstrating adept self‐regulation (Roche et al. 2013), Dark Triad self‐monitoring (Kowalski et al. 2018), positive belief systems (Joshanloo 2021; Zhao et al. 2016), and self‐confidence (O'Reilly and Pfeffer 2021). The state self‐concept often prompts Dark Triad individuals to regulate their behavior effectively (Doerfler et al. 2021), drawing on attributes such as strength use (Sanecka 2021), mental toughness (Papageorgiou et al. 2019), resilience (Szabó et al. 2022), and psychological capital (Zhu and Geng 2023). Dark Triad traits are often associated with self‐monitoring, self‐confidence, or self‐regulatory tendencies. These findings should be interpreted cautiously because such characteristics may vary across traits, individuals, and situations and do not necessarily imply effective or socially constructive regulation. Moreover, the constant desire for self‐assurance in Dark Triad individuals fosters a strong bond with their environment, which supports narcissistic self‐perceived attractiveness (Leslie‐Evans et al. 2022), popularity (Czarna et al. 2016), and effective impression management skills in their social interactions (Ji et al. 2023).

Regarding emotional characteristics, some studies suggest that narcissism is associated with emotional intelligence (Schade et al. 2021) and emotional regulation (Loeffler et al. 2020). Leniarska and Zajenkowski (2022) explain that some of the Dark Triad's emotional strengths stem from being less fearful and less stressed in work‐related tasks. Although personal factors such as loneliness and age‐related psychological effects do not affect the Dark Triad (Carter and Douglass 2018), lower neuroticism may subsequently result in higher psychological and subjective well‐being within the Dark Triad (Aghababaei and Błachnio 2015; Joshanloo 2021). However, these associations are not uniform across traits or settings, and apparent adaptability or social effectiveness should not be equated with prosocial conduct or broader organizational benefit.

On the behavioral side, the adaptive quality of the Dark Triad is associated with greater tendencies toward adaptive behaviors and higher adaptive performance (Grijalva and Harms 2014; Smith and Webster 2018). In addition, the ability to manage uncertain and emergent situations (Smith et al. 2018) helps Dark Triad members accomplish their performance goals and achieve task performance (Fernández‐del‐Río et al. 2020; Nguyen et al. 2021; O'Reilly and Pfeffer 2021), contextual performance (AL‐Abrrow et al. 2020; Liu et al. 2023; Webster and Smith 2019), change‐oriented OCB (Lang et al. 2022), team performance (Nassif 2019), organizational performance (Haar and de Jong 2023), and business performance (Kollmann et al. 2019).

Another generative side of behavioral outcomes that has attracted researchers' attention is the creative side of Dark Triad behaviors. Ji et al. (2023) explored the strong relationship between the Dark Triad and creative performance. Kapoor (2015) and Zhang and Cui (2022) further assert that narcissism is linked to creativity and creative deviance. High risk‐taking abilities (Crysel et al. 2013; Zhao et al. 2016) support the idea that Dark Triad traits are less threatened by the environment and capable of developing innovative outcomes. Narcissistic individuals are highly innovative (Smith and Webster 2018) and craft their jobs (Roczniewska and Bakker 2016) according to job demands. The Dark Triad's creative side also helps them get ahead in their careers (Rovelli and Curnis 2021) and achieve career success (Furnham and Treglown 2021; Spurk et al. 2016).

Another side of a Dark Triad personality is the ability to form connections with groups and people, reflecting a social behavioral dimension. The pioneering studies of the Dark Triad have fully explained narcissistic extroversion. The strong desire of Dark Triad individuals to be popular (Czarna et al. 2016), attractive (Leslie‐Evans et al. 2022), and to manage impressions (Ji et al. 2023) strongly involves them in being committed to social relationships. This may be the reason that Dark Triad individuals have been shown to display highly social behaviors (Robertson et al. 2016) and be highly committed to people at the professional and organizational levels (Cesinger et al. 2023; Kaufmann et al. 2021; Szabó et al. 2023). Grover and Furnham (2021) further emphasize the need to explore and find a positive connection between prosocial behaviors and the Dark Triad. Overall, the Dark Triad generates highly cooperative behaviors (Malesza 2020).

Building on previous research on the emerging positive side of the Dark Triad, Figure 2 provides a comprehensive overview of empirical research on the positive outcomes associated with the Dark Triad.

**FIGURE 2:**
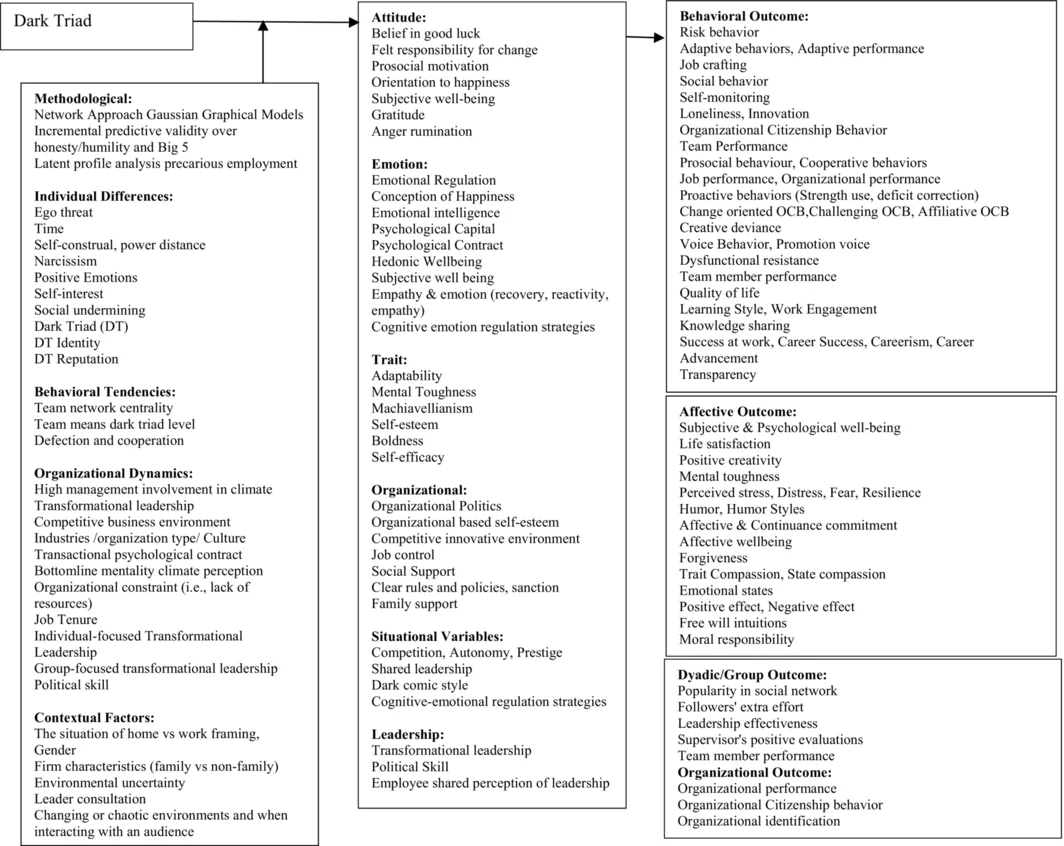
Nomological network of the positive dimensions of the dark triad.

### 4.1 Mediators

Literature explores various mediators that explain the bright outcomes. A distinct pattern emerges from the synthesis of the literature, which broadly categorizes mediators as internal and external mechanisms that generate positive outcomes of the Dark Triad. With respect to internal mechanisms that trigger the positive side of the Dark Triad, Jonason, Wee, and Li (2015) suggested that Dark Triad traits serve as psychological mechanisms that benefit individuals in circumstances such as autonomy, prestige, and competition, ultimately influencing job satisfaction. Zhao et al. (2016) indicated that belief in good luck mediates the relationship between DT traits and risk behaviors, highlighting how irrational beliefs influence risk intentions. Smith and Webster (2018) explored how adaptability mediates the positive impact of grandiose narcissism on innovation in the workplace. Walsh et al. (2020) investigated the positive association between the Dark Triad and emotional regulation, particularly deep acting and emotional labor, affecting job satisfaction. Overall, research by Elbers et al. (2023) and Schade et al. (2021) shows that psychological capital and emotional intelligence, respectively, include factors like adaptability and regulation, which are considered internal but change over time and would help manage individuals' Dark Triad toward positive outcomes. On the other hand, previous studies have investigated the internal self‐stable structures of the Dark Triad, such as mental toughness (Denovan et al. 2021), wellbeing (Womick et al. 2019), self‐esteem (Carnevale et al. 2018), identity (Schyns et al. 2023), and self‐efficacy (Khan et al. 2021; Wang et al. 2023), which predict a strong core of the Dark Triad, the ability to cope with stress and challenges, and one's ability to succeed in specific situations or accomplish a task.

Moreover, external mechanisms have contributed to positive outcomes among Dark Triad members. Mediators such as job control, social support (Baka 2018), family support (Liu et al. 2021), organizational politics (O'Reilly and Pfeffer 2021), rules, policies, and sanctions (Laurijssen et al. 2024), peer behavior, and managerial conduct (Stelmokienė and Vadvilavičius 2022) have been shown to explain constructive approaches to achieving positive outcomes for Dark Triad members through external mechanisms.

Exploring the dynamics of psychological phenomena reveals that many aspects of human experience are shaped by a complex interplay of internal and external factors. For instance, affective and subjective well‐being epitomize this intricate relationship, embodying a blend of internal emotional states and external life satisfaction. Studies shed light on this connection, revealing how individuals' emotional regulation styles and self‐esteem levels interact with external factors to influence their overall well‐being (Gómez‐Leal et al. 2023; Mojsa‐Kaja et al. 2021; Womick et al. 2020). Similarly, the psychological contract is a combination of internal cognitive processes and external environments; it arises from beliefs about exchange relationships and an understanding of the mutual responsibilities of the employee and employer (Zagenczyk et al. 2014). Likewise, shared leadership, another crucial aspect of human experience, emerges as a fusion of beliefs that are not confined to individuals but distributed across team members. Nassif (2019) bridges the literature on social network analysis, team leadership, and the Dark Triad by examining how shared leadership influences team performance.

Finally, motivation to lead, a significant driver of organizational behavior, emerges from the intersection of the leader's internal perception and external leadership or cultural influences.

Kennedy et al. (2021) emphasized both individual motivation and external factors, such as leadership opportunities and cultural norms, in shaping individuals' aspirations to lead. Specifically, they identified affective motivation to lead and social normative motivation to lead (the extent to which an individual treats leadership as a responsibility and duty in an organizational environment) (Kennedy et al. 2021).

### 4.2 Moderators

The moderators in the study span a wide array of dimensions, including methodological approaches, individual differences, behavioral tendencies, organizational dynamics, and contextual factors. Methodologically, researchers employ techniques such as network analysis, latent profile analysis, and controlling for Big Five personality traits to examine relationships and patterns in their data (Czarna et al. 2016; Paleczek et al. 2018; Papageorgiou et al. 2019). These approaches provide a structured framework for understanding the complex interplay between Dark Triad traits and outcomes.

On the individual differences front, moderators such as ego threat, narcissism, age, and gender illuminate the unique psychological makeup of individuals and how these factors shape behavior and outcomes (Carter and Douglass 2018; Crysel et al. 2013; Grijalva and Harms 2014; Zamora et al. 2022). Behavioral moderators examine actions and reactions, including deflection and cooperation, political skill, and deceptive situations, highlighting the nuanced ways individuals navigate social and professional interactions (Ji et al. 2023; Kollmann et al. 2019; Lazreg and Lakhal 2022; Malesza 2020).

Organizational moderators highlight how workplace environments, management styles, and firm characteristics influence employee behavior and performance (Webster and Smith 2019; Rovelli and Curnis 2021; Lang et al. 2022). From high management involvement to competitive business environments, these factors shape organizational culture, employee engagement, and overall productivity.

Taken together, Dark Triad traits can facilitate career success, but other evidence shows limited incremental value beyond the Big Five. Across the three Dark Triad traits, narcissism can facilitate creativity, career success, and organizational outcomes, but its effects can turn negative depending on leadership, reputation, self‐esteem, or situational conditions (Hutter et al. 2015; Rovelli and Curnis 2021; Smith and Webster 2018; Szabó et al. 2023; Volmer et al. 2016). Moreover, political skill can improve performance, yet Machiavellianism is also associated with social undermining and counterproductive behavior (Castille et al. 2017; Smith and Webster 2018). Therefore, not all moderators operate in the same direction. Some conditions activate positive behaviors, whereas others buffer or suppress negative behaviors. Thus, there is no single moderator pattern across all three traits. This review shows that positive and negative effects coexist for all three Dark Triad traits.

### 4.3 Review of Theories Used in Dark Triad

There is a lack of a well‐developed conceptual and theoretical framework on the Dark Triad (Harms et al. 2024; Ingold et al. 2015) to guide how the Dark Triad can be beneficial in the workplace (Ingold et al. 2015). Research should examine the Dark Triad in relation to both positive and negative work, paying attention to intervening mechanisms and boundary conditions. Few popular studies have supported the idea that the Dark Triad has positive aspects (Koehn et al. 2019). However, Hogan and Hogan (2001) suspect that the Dark Triad has long‐term problems associated with it that make these traits less likely. However, every trait has a bright and a dark side, which may be activated due to the underlying mechanism that operationalizes it (Spain et al. 2014).

In personality research, scholars have focused on a limited set of theories, including Trait activation theory, social exchange theory, and socio‐analytic theory (Smith et al. 2018). A theory can offer a different perspective, identify several moderators, and yield multiple implications, providing insight into personality from a different lens (Smith et al. 2018). It may not only advance the field of personality but also reveal new phenomena. Therefore, there is a strong need to study the field of personality using different theories to advance understanding of both the bright and the dark sides of personality (Smith et al. 2018). Apart from that, few new theories have emerged to study the upside of the Dark Triad. Therefore, this research reviews and summarizes the theories that have been used so far in the field of the Dark Triad, as tabulated in Table 1.

**TABLE 1:** Common theoretical perspective for the positive side of dark triad across 107 studies.

Conceptual perspective	Theories represented	Dark triad	Narcissism	Machiavellianism	Psychopathy
Trait and personality perspectives	Life History Theory, Eysenck Theory, Self‐Construal Theory; Reinforcement Sensitivity Theory, Personality Theory; Authenticity Framework	4	2	2	1
Motivational and self‐regulatory perspectives	Self‐Determination Theory, Threatened Egotism Theory, Regulatory Focus Framework, Public Service Motivation Theory, Construction Theory	3	2	1	1
Social and relational perspectives	Social Exchange Theory, Social Learning Theory, Social Influence Theory, Social Interdependence Theory, Social Network Theory, Attachment Theory, Relational Identity Theory, Mimicry Deception Theory, Theory of Mind	2	2	4	3
Situational and contextual perspectives	Trait Activation Theory; Situation Theory, Person–Organization Fit Theory, Job Demands Theory, Conservation of Resources Theory, Theory of Planned Behaviour, Economic Game Theory	5	2	5	1
Leadership and organizational perspectives	Dominance Complementary Theory, Transformational Leadership Theory, Upper Echelon Theory, Agency Theory, Psychological Contract Theory, Leadership Theory, Ethical Leadership, Role Set Theory	3	1	3	2
Cognitive, behavioral and moral perspectives	Theory of Impression Management, Differential Association Theory, Callousness Theory of DT	1	1	2	3

Little attention has been paid to the theoretical architecture underlying DT research. At first glance, the literature appears theoretically diverse, drawing on evolutionary theory, life history theory, social cognitive theory, social exchange theory, trait activation theory, self‐determination theory, social identity theory, conservation of resources theory, regulatory focus theory, resource control theory, and numerous other perspectives (Bandura 1986, 1997; Blau 1964; Griskevicius et al. 2011; Jonason and Webster 2010; Hobfoll 1989; Ryan and Deci 2000; Tajfel and Turner 2004; Tett and Burnett 2003). Theories repeatedly converge on a relatively small set of underlying mechanisms. Consequently, the apparent theoretical diversity of the field masks conceptual similarity.

The first perspective concerns the remarkable dominance of adaptation‐based explanations. Evolutionary theory and life history theory have become the dominant framework for explaining *why narcissism, Machiavellianism, and psychopathy exist* (Paulhus and Williams 2002; Jonason et al. 2009; Figueredo et al. 2006; Kaplan and Gangestad 2015). Regardless of the context, these theories portray Dark Triad traits as strategic adaptations that maximize resource acquisition, status, mating opportunities, or survival under environmental uncertainty. These perspectives have substantially advanced our understanding of the evolutionary origins of socially aversive personalities.

The second perspective draws on the behavioral consequences of the Dark Triad. This perspective encompasses social learning theory, social exchange theory, the theory of planned behaviour, agency theory, job demands theory, human capital theory, person–organization fit theory, psychological contract theory, upper echelons theory, leadership theories, ethical leadership, public service motivation theory, social network theory, role set theory, and related perspectives. Although these theories originate from different disciplines, they share a common assumption that *behavior is shaped through interactions between individuals and their social or organizational environments*. For instance, social cognitive theory proposes that Dark Triad individuals regulate their behavior through cognitive evaluations, efficacy beliefs, observational learning, and reciprocal interactions with their environment (Hafeez et al. 2025). Social exchange theory assumes the relational aspect that individuals possessing Dark Triad characteristics strategically evaluate interpersonal relationships based on anticipated costs, benefits, and expected returns. However, the mechanisms differ; both theories ultimately portray individuals high in Dark Triad traits as rational actors who strategically adjust their behavior to maximize personal gains.

A third pattern reveals the psychological processes underlying Dark Triad traits. Trait theories such as the five‐factor model and HEXACO primarily describe *who Dark Triad individuals are* (Costa Jr. and McCrae 2010; Lee and Ashton 2005). Evolutionary perspectives explain *why* the *Dark Triad emerged* (Jonason et al. 2009). In contrast, behavioral theories including social cognitive theory, trait activation theory, conservation of resources theory, and self‐determination theory attempt to explain *when, where, and how these traits influence behavior* (Bandura 1986; Hobfoll 1989; Ryan and Deci 2000; Tett and Burnett 2003). Therefore, rather than competing with one another, theories occupy different explanatory levels within the same nomological network. Ironically, this layered theoretical structure has rarely been acknowledged explicitly in the Dark Triad literature.

Consequences of the Dark Triad are most commonly studied in research than the Dark Triad itself. For instance, social exchange theory explains manipulation. Social cognitive theory explains efficacy and behavioral regulation (Hafeez et al. 2025). Social identity theory helps to explain group identification phenomena (Tajfel and Turner 2004). Conservation of resources theory explains how individuals manage their resources and reduce stress (Hobfoll 1989). Similarly, trait activation theory helps explain when and why personality expressions occur in a given context (Tett and Burnett 2003). All suggest that the majority of the existing literature about the Dark Triad is using an outside framework to provide an explanation as to why people exhibit certain behaviors. This would indicate that the work being done with respect to the Dark Triad is becoming much more focused upon identifying specific outcomes related to those behaviors and less concerned with developing original explanations for *what causes them*.

All three perspectives raise the following research question for future research. *Has the field is heading to develop new theoretical insights into the Dark Triad, or it repeatedly applied different theoretical lens to explain fundamentally similar psychological processes?* These theories appear to point to identical explanatory mechanisms, suggesting that true theoretical pluralism may exist in name only. Thus, researchers need to look beyond simply applying well‐established behavioral theories and focus on creating more comprehensive frameworks that outline the unique ways in which Dark Triad traits produce distinct cognitive, motivational, and behavioral outcomes. An integrative approach would not only reduce conceptual redundancy but also advance a more coherent theoretical foundation for Dark Triad research.

## 5 Future Research Directions

Personality relationships are far more complex than suggested (Smith et al. 2018). Therefore, scholars should move from simple personality associations to the underlying mechanisms that form and drive these relationships. Understanding the Dark Triad itself and its underlying mechanisms through diverse theories is required. For instance, Research has merely focused on the explicit side of personality, namely traits (LeBreton et al. 2018). The implicit side (i.e., motives, beliefs, unconscious, implicit biases) of personality is needed to explore the Dark Triad relationship with work outcomes (LeBreton et al. 2018). Models that capture both explicit and implicit aspects of the Dark Triad will provide greater insight into how personality relates to and predicts behavior (LeBreton et al. 2018). Moreover, future research should prioritize theory integration and comparative testing of competing theoretical explanations to develop a more coherent understanding of the conditions under which Dark Triad traits facilitate positive organizational outcomes.

This research opens many avenues for further study such as the psychological mechanisms that can alter the negative relationship between the Dark Triad traits and employee work outcomes. This review suggests that future research should use diverse theoretical perspectives to examine the psychological mechanisms underlying the Dark Triad traits. Future researchers could explore additional aspects of these mechanisms to examine the relationship between the Dark Triad and performance. A growth mindset may provide new possibilities for Dark Triad research. For instance, the Dark Triad can be examined through the lens of incremental theories to determine which Dark Triad traits are malleable. Malleable traits that can adapt to situations are more likely to adjust and perform well in a fast‐paced environment (Tasselli et al. 2018). Furthermore, in a high‐technology and rapidly changing environment, it is worth investigating whether it is better to recruit individuals with strong, rigid traits or to hire individuals with malleable traits that change with the needs of the environment (Tasselli et al. 2018), irrespective of whether it is the Dark Triad traits or the Big Five personality triad. For instance, all three Dark Triad traits are expected to be more malleable due to high self‐monitoring and self‐regulation tendencies (Kowalski et al. 2018; Roche et al. 2013), which leads to predicting that they can also learn and develop their skills from the environment.

Another possible angle is to study personality from a bi‐directional angle and view personality as an outcome rather than as an antecedent (Dalal et al. 2020). None of the 107 studies examined personality as an outcome. For instance, social cognitive theory offers another theoretical angle, assuming that behavior is shaped by both personal and environmental factors, and that motivational and self‐regulatory mechanisms direct that behavior, which in turn affects and is affected by the environment in bi‐directional ways, further influencing personality and the environment. Table 2 presents the proposed directions for future research.

**TABLE 2:** Avenues for future research.

Future research domain	Research questions within the domain	How to investigate research questions
Mediators/Moderators	What conditions, situations, and mechanisms may strengthen, dampen, amplify, and attenuate the relationship of the dark triad with negative work outcomes?	Use the ad hoc and idiosyncratic conceptualization of situations where situational taxonomies of events may help to understand the positive aspects of dark personality. Such as Joshanloo (2021) has used the concept of happiness as a mechanism to alter the traditional negative relationship between the dark triad and well‐being.
Antecedent of dark triad	What are the motives and drives of a dark personality?	The motivational framework provides researchers with a way to conceptualize individual differences (Jonason and Zeigler‐Hill 2018). Use various affiliative and psychological motives to understand the dark triad. A study by Jonason and Zeigler‐Hill (2018) used social motives to understand the fundamentals and predictors of the dark triad. Other research directions could be Such as narcissists' need for external approval to drive them to more creative and innovative and hubristic individuals' irresistible self‐belief enables them to work on idea‐generation tasks despite constant setbacks (Smith et al. 2018).
Bright core	Which traits of the dark triad have bright facets that illuminate the bright side of the dark triad? What traits among the dark triad shared the common and overlapping variance? What is the nomological network of the dark triad and how it could be extended to a positive criterion?	Design studies that could measure the shared variance and common core of the dark triad. Extend the criterion of the dark triad to core self‐evaluation, self‐esteem, and life satisfaction (Volmer et al. 2019). Use data analytic techniques such as the bi‐factor model (Volmer et al. 2019) factorial structure (Bertl et al. 2017), and hierarchal structure to measure the key features and bright core of the dark triad.
Methodological	To what extent does reliance on self‐report data develop a substantial conclusion? How to improve the precision of self‐report data (e.g., use of experience sampling designs)? To what extent does the difference in self‐versus peer/supervisor report data reflect various explanations?	Peer and supervisor‐rated surveys could be used as these are the best source to report and measure dark personality (Muris et al. 2017; Jonason and Zeigler‐Hill 2018; Volmer et al. 2019) Experience sampling designs could be used to measure personality consistency and avoid same source bias and high validity (Green et al. 2019).
Novel theories	What are the theories that can explain the brighter aspects of dark personality?	Increase the precision of theories in the dark triad field by applying novel theories that give a brighter and more positive insight into dark personality. Such as social cognitive theory (Smith et al. 2018) which may assume that any dark tendencies could be avoided by social learning, observing, and enactive mastery.
Multilevel	How do team and group‐level variables exacerbate and dampen the effect of the dark triad on group outcomes? What is the relationship between dark triad and work outcomes within the team? How firm level variables inhibit the deviant behaviors of dark personalities? How dark personality be constrained to positively influence firm‐level performance?	Investigate team‐level variables such as firm policies, organizational culture, and group team norms that could inhibit deviant dark behaviors at team, group, and firm levels (Smith et al. 2018)

Third, relatively little attention has been devoted to individual Dark Triad trait dimensions. Across the 107 studies, Dark Triad traits have been studied as a composite construct or primarily focused on narcissism, with comparatively fewer studies examining how Machiavellianism or psychopathy influence positive workplace outcomes (Grijalva and Harms 2014; Ji et al. 2023; Smith and Webster 2018). Therefore, the present review provides a stronger understanding of narcissism than of the remaining Dark Triad dimensions.

Moreover, the literature on the positive organizational outcomes remains limited. Existing research focuses on OCB, career success, creativity, innovation, leadership effectiveness, and employee well‐being (Lang et al. 2022; Liu et al. 2023; Spurk et al. 2016; Webster and Smith 2019). However, relatively little attention has been devoted to emerging organizational outcomes such as digital collaboration, AI‐assisted decision‐making, machine learning, virtual leadership, sustainable performance, management, organizational resilience, and strategic decision quality. The narrow focus restricts the broader understanding of how DT traits operate within rapidly evolving organizational environments.

The methodological limitations are consistent across all the research. Most studies used cross‐sectional surveys that relied on participants' self‐reports to assess their attitudes. A very few studies have used experimental design methods (Crysel et al. 2013; Zamora et al. 2022), longitudinal designs (Zhu and Geng 2023), multiple assessment sources (Szabó et al. 2023), latent profile analysis (LPA) (Nguyen et al. 2021), and network analysis (Papageorgiou et al. 2019) to explain the causality for how dark triad personality traits influence work‐related outcomes as a function of development. As a result, the lack of empirical evidence on psychopathy is relatively small compared with that for other dark triad traits. Furthermore, much of this research has produced conflicting results. For example, while successful psychopathy, especially boldness, has been linked to effective leadership, subjective well‐being, and job satisfaction (Du and Templer 2022), other psychopathic characteristics continue to predict abusive supervision, reduced voice behavior, and unethical decision‐making (Laurijssen et al. 2024; Zhuang et al. 2022). These inconsistencies suggest that future research should distinguish between psychopathy subdimensions rather than treating psychopathy as a homogeneous construct.

## 6 Conclusion

The present study reviewed 107 studies involving data from 51,124 individuals and several methods to examine the conditions under which Dark Triad traits may be associated with favorable organizational outcomes, alongside their potential harms. The findings highlight the need for context‐sensitive explanations that account for potential work outcomes and the ethical and interpersonal consequences of Dark Triad traits. It may be opportune to reconsider the prevailing approach, revise judgments, and reimagine the labels, research, and educational paradigms to better address the multifaceted nature of the Dark Triad and its implications.

## Funding

The authors have nothing to report.

## Ethics Statement

The authors have nothing to report.

## Consent

The authors have nothing to report.

## Conflicts of Interest

The authors declare no conflicts of interest.

## Supporting information


**Appendix A:** Key search terms of study with Google Scholar results.
**Appendix B:** Review of positive outcomes of dark triad.

## Data Availability

Data sharing not applicable to this article as no datasets were generated or analysed during the current study.
