# Supplementary material for: When and Which Dark Traits Shine? A Multidimensional Review of the Dark Triad in Organizational Research
Source: Psych J. 2026 Nov 13;15(6):e70130. doi: 10.1002/pchj.70130 (PMC13768806; doi:10.1002/pchj.70130)
Supplement: Supplementary file 1 [file PCHJ-15-e70130-s001.docx]

**APPENDIX**

**When and Which Dark Traits Shine? A Multidimensional Review of the Dark Triad in Organizational Research**

**Table of Contents**

APPENDIX A. Key Search Terms of Study with Google Scholar results ………………….3

APPENDIX B. Review of Positive Outcomes of Dark Triad……………………………….5

| **APPENDIX A** Key Search Terms of Study with Google Scholar results | | | |
| --- | --- | --- | --- |
| **Dark Triad** | **Narcissism** | **Machiavellianism** | **Psychopathy** |
| Advantages of Dark Triad Characteristics: 4,520 | Advantages of Narcissistic Personalities: 854 | Advantages of Machiavellian Leadership: 21,200 | Advantages of Psychopathic Personalities: 1200 |
| Advantages of Embracing the Dark Triad: 327 | Advantages of Narcissistic Strategies: 46 | Advantages of Machiavellian Personalities: 15,400 | Advantages of Psychopathic Strategies: 1 |
| Advantages of Leveraging Dark Triad Strategies: 813 | Benefits from Narcissistic Traits: 3,370 | Benefits from Machiavellian Attributes: 21,700 | Benefits from Psychopathic Traits: 7,560 |
| Benefits from Dark Triad Attributes: 4,210 | Benefits of Narcissistic Behavior: 1,690 | Benefits of Machiavellian Behavior: 401 | Benefits of Psychopathic Behavior: 48 |
| Benefits of Cultivating Dark Triad: 2,440 | Benefits of Narcissistic Mindset: 56 | Benefits of Machiavellian Personality Traits: 17,900 | Benefits of Psychopathic Mindset: 19 |
| Benefits of Dark Triad: 11,000 | Benefits of Narcissistic Personality Traits: 2,140 | Benefits of Machiavellian Planning: 28,600 | Benefits of Psychopathic Personality Traits: 2,440 |
| Benefits of Integrating Dark Triad Tactics: 7,020 | Bright Outcomes of Narcissistic Decision-Making: 8 | Bright Outcomes of Machiavellian Decision-Making: 4 | Bright Outcomes of Psychopathic Decision-Making: 4 |
| Beneficial Attributes of the Dark Triad: 93 | Bright Outcomes of Narcissistic Self-Perception: 2,340 | Bright Outcomes of Machiavellianism: 12 | Bright Outcomes of Psychopathic Tendencies: 1 |
| Bright Outcomes from Dark Triad Mindset: 3 | Brighter Results of Narcissistic Thinking: 1 | Brighter aspects of Machiavellian Calculations: 11 | Brighter Results of Psychopathic Thinking: 1 |
| Bright Outcomes of Dark Triad Behaviors: 14 | Brighter Side of Narcissism: 386 | Brighter Side of Machiavellianism: 148 | Brighter Side of Psychopathy: 107 |
| Brighter Prospects with Dark Triad Qualities: 58 | Favorable Consequences of Narcissism: 186 | Favorable Consequences of Machiavellian Actions: 54 | Favorable Consequences of Psychopathic Action: 217 |
| Brighter Results from Dark Triad Tendencies: 2 | Favorable Consequences of Narcissistic Actions: 154 | Favorable Consequences of Machiavellian Characteristics: 149 | Favorable Consequences of Psychopathy: 52 |
| Brighter Side of Dark Triad leadership: 1,770 | Favorable Outcomes from Narcissistic Behavior: 564 | Favorable Outcomes from Machiavellian Behavior: 258 | Favorable Outcomes from Psychopathic Behavior: 555 |
| Favorable Features of the Dark Triad: 5 | Gains Arising from Narcissistic Behaviors: 293 | Gains Arising from Machiavellian Strategies: 14,400 | Gains Arising from Psychopathic Behaviors: 143 |
| Favorable Outcomes of the Dark Triad: 221 | Gains from Narcissistic Adaptability: 3,830 | Gains from Machiavellian Adaptability: 2,630 | Gains from Psychopathic Adaptability: 1,120 |
| Favorable Outcomes from Dark Triad Interactions: 192 | Gains from Narcissistic Dispositions: 19,200 | Gains from Machiavellian Calculations: 35 | Gains from Psychopathic Dispositions: 3,330 |
| Favorable Results of Dark Triad Actions: 1,060 | Gains from Narcissistic Leadership: 641 | Gains from Machiavellian Mindset: 3,070 | Gains from Psychopathic Leadership: 98 |
| Positive Aspects of the Dark Triad: 943 | Positive Effects of Narcissistic Tendencies: 587 | Positive Effects of Machiavellian Strategies: 2,730 | Positive Effects of Psychopathic Tendencies: 366 |
| Positive Consequences of the Dark Triad: 541 | Positive Outcomes of Narcissistic Traits: 731 | Positive Outcomes of Machiavellian Strategies: 2,290 | Positive Outcomes of Psychopathic Traits: 449 |
| Positive Consequences of Embodying the Dark Triad: 652 | Pros of Narcissistic Approaches: 17,400 | Pros of Machiavellian Approaches: 5,730 | Pros of Psychopathic Approaches: 3,030 |
| Positive Effects of Dark Triad Characteristics: 1,890 | Pros of Narcissistic Traits: 7,620 | Pros of Machiavellian Traits: 2,620 | Pros of Psychopathic Traits: 603 |
| Positive Impact of Dark Triad Traits on Performance: 1,560 | Prospects of Narcissistic Features: 173 | Prospects of Machiavellian Approaches: 18,400 | Prospects of Psychopathic Features: 250 |
| Pros of Dark Triad Personalities: 889 | Upsides of Narcissistic Attributes: 119 | Qualities of Machiavellian: 23 | Upsides of Psychopathic Attributes: 42 |
| Upsides of Dark Triad Behaviors: 296 | Upsides of Narcissistic Characteristics: 24 | Upsides of Machiavellian Dispositions: 25 | Upsides of Psychopathic Characteristics: 108 |
| Upsides of Dark Triad Personality Traits: 224 | Upsides of Narcissistic Influence: 736 | Upsides of Machiavellian Influence: 222 | Upsides of Psychopathic Influence: 518 |
| Upsides of Developing Dark Triad Attributes: 395 | Upsides of Narcissistic Mindset: 206 | Upsides of Machiavellian Mindset: 58 | Upsides of Psychopathic Mindset: 118 |
| Upswings in Achievement due to Dark Triad Characteristics: 3 | Upswings of Narcissistic Attributes: 161 | Upsides of Machiavellian Persona: 223 | Upsides of Psychopathic Persona: 150 |
| Upswings of Dark Triad Traits: 153 | Upswings of Narcissistic Characteristics: 87 | Upsides of Machiavellian Tactics: 128 | Upsides of Psychopathic Tendencies: 35 |
| Upswingsof Dark Triad Characteristics: 787 | Upswings of Narcissistic Tendencies: 563 | Upswings of Machiavellian Dispositions: 25 | Upswings of Psychopathic Attributes: 38 |
| Upswings of Embracing the Dark Triad: 725 | Upswings of Narcissistic Personalities: 1,290 | Upswings of Machiavellian Thinking: 67 | Upswings of Psychopathic Characteristics: 30 |
| (after removing overlapped articles)  Total articles: 11,723 | Total articles: 8,202 | Total articles: 5,603 | Total articles: 5,313 |
| Total: 12,840 (after removing overlapped articles) | | | |

Note. Search date 22-4-25 to 27-8-25

**APPENDIX B** Review of Positive Outcomes of Dark Triad

| Source | sample | Predictor | Mechanism/Situation/Condition | | Criterion tested | Guiding Theories | Findings/ Theoretical Contribution |
| --- | --- | --- | --- | --- | --- | --- | --- |
|  |  |  | Condition/moderator | Mechanism/Mediation |  |  |  |
| Roche et al, 2013  **Journal of Personality Assessment** | 5383  (Students) | N |  |  | Self-regulation |  | Integrates both pathological and normal narcissism, structured around mechanisms of self-regulation. |
| Crysel et al, 2013  **Personality & Individual difference** | 1404 (students) | DT | Ego threat |  | Risk behavior | Life history theory | Links the Dark Triad with impulsivity, sensation-seeking, gambling, and discounting behaviors. Additionally, experimental manipulation of ego threat sheds light on causal processes connecting narcissism and temporal discounting. |
| Grijalva & Harms 2014  **Academy of Management perspective** | 3124 | N | Changing or chaotic environments and when interacting with an audience |  | Adaptive behaviors, Adaptive performance | Dominance complementary theory, Theory of threatened egotism, and aggression | Narcissism-CWB relationship, suggest organizational interventions and introduce the Narcissistic Leaders and Dominance Complementarity Model for follower compatibility. |
| Aghababaei & Balachino, 2015  **Personality & Individual difference** | 220 | N |  |  | Subjective Wellbeing,  Psychological well-being, life satisfaction |  | Dark Triad traits to both eudaimonic and hedonic well-being  Top of Form |
| Kapoor, 2015  **Creative Research Journal** | 51 | N |  |  | Positive creativity |  | Measuring creativity through implicit association test |
| Jonason et al,2015a  **Personality & Individual difference** | 361 | DT |  | Competition, Autonomy, Prestige | Job satisfaction |  | View dark triads as psychological mechanisms that benefit in circumstances such as autonomy, prestige, and competition |
| Spurk et al, 2015  **Social Psychology and Personality Science** | 793 | N |  |  | Objective and Subjective Career Success |  | Dark Triad's role in explaining important career outcomes. Implications for personality and career research are derived |
| Czarna et a.,l 2016  **Personality and Social Psychology Bulletin** | 273 | N | Time |  | Popularity in social network | Social network theory | Dark triad fame and popularity over longer time frames using inferential network analysis |
| Roczniewska& Bakker, 2016  **Journal of Psychology** | 155 | DT |  |  | Job crafting | Eysenck theory, Job demands theory | Demonstrate two distinct personality constructs—PEN and the Dark Triad—as predictors of job-crafting behaviors |
| Robertson et al-2016  **Personality & Individual difference** | 469 | DT | Self-construal, power distance |  | Social behavior | Self-construal theory | Dark Triad traits operate differently across the countries due to differences in the cultural variables of self-construal and power distance. |
| Zhao et al,2016  **Frontier in Psychology** | 777 | DT |  | Belief in good luck | Risk behaviors (corrupt intention) | Theory of planned behavior | Dark Triad tendencies are more likely to engage in corruption but also support the role of their irrational beliefs in good luck |
| Kowalski et al, 2018  **Personality & Individual difference** | 244 | DT |  |  | Self-monitoring |  | Examining the associations between the Dark Triad and dimensions reflecting types of self-presentation, specifically, socially desirable responding and self-monitoring |
| Carter & Douglass, 2018  **Frontier in Psychology** | 200 | Age | Narcissism |  | Loneliness |  | Narcissism potentially benefits age-related loneliness and psychological well-being. |
| Smith & Webster, 2018  **Personality & Individual difference** | 181 | N |  | Adaptability | Innovation |  | Grandiose narcissism positively impacts employee innovation |
| Kollmann et al., 2018  **Journal of Small Business Management** | 132 | EC | Narcissism |  | Business Planning performance | Human capital theory | Develops Positive task conflict performance link  Potential positive aspect of narcissism in the team context |
| Webster & Smith,2019  **Journal of Business and Psychology** | 298 | N | High management involvement in climate |  | Organizational Citizenship Behavior | Self-determination theory | Used SDT to address the needs of Dark Triad traits in the organizational environment |
| Papageorgiou et al,2019  **Journal of Personality** | 748 | DT | Network Approach (Gaussian Graphical Models) |  | Mental toughness. perceived stress |  | Focusing on the dynamic continuum of personality and perceiving narcissism as beneficial rather than a dichotomous way of perceiving personality |
| Fernández-del-Río et al-2019  **Personality & Individual difference** | 613 | DT | incremental predictive validity over honesty/humility and Big 5 |  | Task performance, contextual performance |  | Contributed to using Dark tetrad positively above the normal range of personality measures such as Big 5 and HEXACO |
| Walsh et al-2019  **Management Decision** | 269 | DT |  | Emotional Regulation | Job satisfaction |  | Explored dark triads' positive connection with deep acting and emotional labor |
| Nassif, 2019  **Human Resource Management Review** |  | DT | Team network centrality, Team means dark triad level | Shared leadership | Team Performance | Social interdependence theory | Contribute by bridging the literature on social network analysis, team leadership, and dark triad |
| Abrrow et al, 2020  **Global Business and Organizational Excellence** | 414 | DT | Positive Emotions |  | Organizational Citizenship Behavior | Agency theory | Explain the role of positive emotions in altering the positive organizational outcomes of the dark triad |
| 2020-Grover & Furnham  **Personality & Individual difference** | 903 | DT | The situation of home vs work framing, Gender |  | Prosocial behavior and work behaviors | Situation Theory | Contributed positively to framing the Dark triad and found no difference between the big five and the dark triad while framing these traits in a work context |
| 2020-Loeffler et al  **Frontier in Psychology** | 60 | N |  |  | Emotion Regulation |  | Contribute to a better understanding of the regulation of emotions in vulnerable and grandiose narcissism. |
| Malesza, 2020  **Current Psychology** | 280 | DT | Defection and cooperation |  | Cooperative behaviors | Economic game theory | Contribute to understanding the dark triad role in defection and cooperation where narcissists found less toward defection |
| Rovelli & Curnis, 2020  **Leadership Quarterly** |  | N | Firm characteristics (family vs non-family firms) |  | Career Advancement | Life history theory | Contribution to explaining narcissist personality in the career advancement of narcissist CEOs |
| Joshanloo-2021  **Frontier in Psychology** | 1177 | DT |  | Conception of Happiness | Well-being |  | DT traits link to distinct views on well-being, suggesting individuals with these traits lean towards specific notions of positive beliefs. |
| Furnham & Treglown-2021  **Frontier in Psychology** | 303 | N |  | Work Engagement | Success at work, Career Success |  | Explain narcissism's relation with adjustment, engagement |
| Schade et al,2021  **Frontier in Psychology** | 749 | DT |  | Emotional intelligence | Cyberbullying |  | Emotional intelligence as a buffer to reduce the negative relationship of grandiose and vulnerable narcissism with cyberbullying |
| Nguyen,2021  **Personality & Individual difference** | 157 | DT | Latent profile analysis | Person-centered approach | Job performance |  | Contributed using latent profile analysis and bi-factor model to study dark triad |
| Kaufmann et al, 2021  **Frontier in Psychology** | 157 | DT | precarious employment (career interruptions and part-time or casual work) |  | Professional Commitment |  | The first study to use precarity developing the relationship while using distinct sub-scales |
| Zhu & Geng 2021  **Current Psychology** | 577 | N |  | Psychological Capital | Health |  | Time-lagged study to explain causal relationships with health |
| Evans et al, 2021  **Personality & Individual difference** | 482 | DT | Gender |  | Self-perceived attractiveness |  | Narcissist men scored higher than women on perceived attractiveness |
| Sanecka, 2021  **Plos One** | 412 | N |  |  | Proactive behaviors (Strength use and deficit correction) | Regulatory focus framework | Contribute to explaining the motivational factors of grandiose and vulnerable narcissism |
| O’Reilly & Pfeffer, 2021  **Personality & Individual difference** | 911 | N |  | Organizational Politics | Performance |  | Grandiosity, self-confidence, entitlement, and exploiting others are viewed as effective tools. |
| Leniarska & Zejankowski-2022  **Frontier in Psychology** | 634 | N |  | Intelligence Self-views | Distress, Fear |  | Narcissist admiration and narcissist rivalry relate positively with distress and intelligence |
| Szabo et al,2022  **Personality & Individual difference** | 518 | DT |  | Mental Toughness | Resilience |  | Contribute to theory by giving positive evidence on the dark triad |
| Zamora et al, 2022  **Personality & Individual difference** | 385 | DT | Self-interest |  | Utilitarian moral decisions |  | Experimentally examine the DT🡪Utilitarianism relation in sacrificial moral dilemmas. |
| Dionigi et al., 2022  **Personality & Individual difference** | 342 | DT |  |  | Humor, Comic style |  | Predict Narcissism with light humor styles and Machiavellianism and psychopathy with lighter style among 8 humor styles |
| Lazreg & Lakhal, 2022  **Acta Psychologica** | 289 | DT | Political skill, Deceptive Situation |  | Exploitative leadership | Trait activation and socio-analytic theory | Use deceptive conditions (situation as a predictor of behavior) to predict exploitative leadership of dark triad |
| Lang et al, 2022  **Frontier in Psychology** | 820 | N | Environmental uncertainty | Felt responsibility for change | Change oriented OCB | Trait activation theory | Narcissism as antecedent of change-oriented OCB |
| Zhang et al., 2022  **Frontier in Psychology** | 654 | N | Transformational leadership | Creative Self-efficacy | Creative deviance | Narcissism theory trait activation theory | Expanding the understanding of creative self-efficacy to understand creative deviance as a positive phenomenon |
| Cesinger et al,2023  **Review of Managerial Science** | 415 | N |  |  | Organizational Commitment | Self-affirmation theory | Contributes to destigmatizing Dark Triad traits and fills gaps in research on manager personality and organizational commitment. |
| Haar & Jong,2023  **Frontier in Psychology** | 523 | DT | Competitive business environment |  | Sales breakthrough, Organizational performance | Upper echelon theory | Contribute to explaining the phenomenon of managerial capital to alter negative dark tendencies |
| Liu et al, 2023  **Current Psychology** | 610 | N |  | Prosocial motivation | Organizational Citizenship behavior |  | Narcissistic individuals when motivated by prosocial values can exhibit positive behaviors that benefit their organization |
| Szabo et al, 2023  **Personality & Individual difference** | 442 | N |  |  | Organizational identification, organizational commitment, and job satisfaction |  | Contribute using multisource data to measure dark personality and its relation with work attitudes and work motivation |
| Stelmokiene et al,2023  **Psihologijske Teme** | 264 | DT | Culture | Transformational leadership | Followers' extra effort, Leadership effectiveness, Satisfaction with the leader | Transformational leadership theory | Explains how negative leader tendencies could be related to positive work outcomes |
| Ji et al, 2023  **Frontier in Psychology** | 530 | N | Political skill | Personal reputation | Creativity | Theory of impression management | Develop an inverted U-shape relationship of Narcissism and creativity at low political skill |
| Howard et al,2022  **Journal of Organizational Effectiveness: People and Performance** | 356 | DT |  | Self-deceptive Enhancement  Subjective invulnerability | Social Courage | Reinforcement sensitivity theory | Narcissism intersects with novel theoretical frameworks, such as those concerning ethical decision-making and self-regulation, alongside social courage. |
| Zagenczyk et al., 2014  **Journal of Management** | 248 | M |  | Psychological Contract | OCB | Psychological contract theory | The influence of Mach on performance is shaped by how employees perceive psychologically their employment relationships |
| Fennimore,2021  **Public Personnel Management** | 312 | N |  | Public Service Motivation (PSM) | Non-profit sector | Public Service Motivation Theory | Links narcissism to PSM's aspects of self-sacrifice, inclination toward public engagement, and compassion dimensions |
| Bulbuc & Pestra, 2024  **Personality & Individual difference** | 380 | DT |  |  | Authenticity  Core self-view | Kernis and Goldman's (2006) framework of authenticity. | High dark triad individuals have agentic, self-interested tendency than people low in DT. |
| Smith & Webster,2018  **Personality & Individual difference** | 425 | M | Social undermining | Political Skill | Job performance | Trait activation theory | Machiavellian traits feel socially undermined by their colleagues, they tend to exhibit greater political skill. This enhanced political skill is associated with better job performance ratings |
| Belschak et al, 2015  **Journal of Management** | 276 | M | Transformational leadership Transactional psychological contract |  | Challenging OCB | Trait activation Theory | increased job autonomy and intrinsic motivation resulting from transformational leadership partially account for the link between transformational leader behavior and challenging citizenship behavior among Machiavellian followers.Top of Form |
| Castille et al,2018  **Journal of Business Ethics** | 368 | M | Bottomline mentality climate perception |  | Prosocial Behavior | Trait activation Theory | Machiavellians exhibit a greater willingness to engage in unethical pro-organizational behavior while perceptions of bottom-line mentality climate may not impact their motivation to do so. |
| Castille et al,2017  **Personality & Individual difference** | 295 | M | Organizational constraint (i.e., lack of resources) | Social undermining | Supervisor's positive evaluations, performance, production deviance | Trait activation Theory | Machiavellist produce competitive environment by undermining their coworkers |
| Zettler & Solga,2013  **European Journal of Personality** | 198 | M | Job Tenure |  | Task performance  Organizational Citizenship behavior | Social exchange theory  Social influence theory | Employees displaying intermediate levels of Machiavellianism exhibited the highest degree of organizational citizenship behavior compared to both lower and higher levels. |
| Blickle et al,2020  **Journal of Vocational Behavior** | 310 | M | Job tenure  Political skill |  | Career role performance  Counterproductive work behaviors | Socioanalytic theory  Mimicry deception theory | A two-study triangular research design involving 1438 participants was utilized. The findings aligned with socioanalytic theory, indicating that political skill masked Machiavellianism. However, the data provided only partial support for the mimicry deception theory |
| Koo & Lee,2021  **Journal of Business Ethics** | 450 | M | Individual focused Transformational Leadership  Group-focused transformational leadership |  | Affective Commitment  Continuance commitment  Organizational citizenship behavior | Transformational leadership theory | To guide Machiavellian employees towards pro-organizational behavior, a group-focused Transformational Leadership (TFL) approach is necessary rather than an individual-focused one. |
| Tariq et al, 2021 | 328 | IM | DT |  | Social Capital  Career Success | Structural holes theory | Propose that dark triad significantly strengthen the relationship of impression management and social capital |
| Mclarty et al., 2022  **Journal of Organizational Behavior** | 384 | DT | DT Identity  DT Reputation |  | Organizational Citizenship behavior | Socioanalytic theory | When both self-perception and coworker perception of the Dark Triad are taken into account, supervisor-rated citizenship declines at an increasingly rapid pace. Dark Triad identity and reputation together offer complete understanding of their relationship with Organizational Citizenship Behavior (OCB). |
| Volmer et al, 2016  **Personality & Individual difference** | 215 | DT |  |  | Career Success (Salary, promotion, career satisfaction) |  | Narcissism turned out the brightest trait that benefit in objective and subjective career success also benefits subordinates well-being. |
| Zhuang et al,2022  **International Journal of Hospitality and Management** | 412 | DT | Intention to leave |  | Voice Behavior |  | Machiavellianism and narcissism are positively correlated with voice behavior, while psychopathy is negatively correlated. Moreover, the intention to leave moderates the relationship between both Machiavellianism and psychopathy with voice behavior. |
| Carnevale et al-2018  **Organizational Behavior and Human Decision Processes** | 305 | N | Leader consultation | Organizational based self-esteem | Employee behavior  (Promotive voice, dysfunctional resistance) | Belongingness theory | Indirect impacts of leader narcissism on the four outcomes through OBSE are notable only when narcissistic leaders neglect to consult with their employees, with these effects becoming insignificant when leader consultation is high. |
| Hutter et al, 2015  **Journal of Management Information Systems** | 267 | M |  | Competitive innovative environment | Innovation,  creative ideas, Success | Theory of mind | Machiavellianism stands out as robust predictors of behavior and success in competitive innovation settings. |
| Baka,2018  **International Journal of Occupational Safety and Ergonomics** | 382 | DT |  | Job control  Social Support | Reduced CWB | Social exchange theory, person organization fit theory | The presence of social support amplifies the moderating impact of job control on the association between Dark Triad traits and counterproductive work behavior (CWB). |
| Laurijssen et al,2024  **Journal of Business Ethics** | 460 | P |  | Clear rules and policies, sanction ability of misconduct, and transparency of behavior | Reduced Self-serving, abusive behavior | Social cognitive theory, Social information theory | Rules, policies and sanctions lowers the destructive leadership effects of primary psychopaths |
| Chiaburu et al, 2011  **Journal of Business Ethics** | 320 | P |  | Exchange ideology | Careerism | Social exchange theory | The strength of an employee’s belief that work effort should depend on treatment by the organization |
| Van Groningen et al., 2021  **Personality & Individual difference** | 388 | DT | Narcissism |  | Well-being |  | Narcissism and particularly grandiose narcissism buffers the dark triad and well-being relationship |
| Ruiz-Palomino et al,2018  **Business Ethics: A European Review** | 340 | UB |  | Machiavellianism | Employee’s ethical intention | Role set theory  Differential association theory  Social learning theory | Aligning individuals high in Machiavellianism with ethical peers can mitigate the detrimental impact of Machiavellian tendencies on ethical intentions. |
| Sutton & Stapleton,2023  **BMC Psychology** | 298 | P |  | Employee authenticity | Employee well being |  | Authenticity has the potential to enhance overall well-being and equip employees with strategies to navigate managerial conduct influenced by maladaptive psychopathic traits. |
| Paleczek et al, 2018  **Journal of Managerial Psychology** | 210 | DT | Control for Big five |  | Career Success  Salary  Leadership position | Socioanalytic Theory | The Dark Triad offers limited additional insights compared to the Big Five personality traits when forecasting salary. |
| Elbers et al,2023  **Administrative Sciences** | 315 | DT |  | Psychological Capital | Employee work-related need satisfaction | Self-determination theory | Psychological capital emerged as a partial mediator in the connection between the dark traits of managers and the fulfillment of employees' basic needs. |
| Liu et al,2021  **Frontier in Psychology** | 580 | DT |  | Family support & Hedonic Wellbeing | Eudaimonic wellbeing |  | Narcissism directly influences eudaimonic wellbeing, whereas Machiavellianism and psychopathy affect it through serial two-mediator models involving family support and hedonic wellbeing. |
| Chang et al,2024  **Personality & Individual difference** | 422 | DT4 |  | Dark comic style | Affective wellbeing |  | Dark comic styles, especially cynicism and sarcasm, are closely tied to the Dark Tetrad traits and affective well-being, influencing both positive and negative emotions within this framework. |
| Seto & Davis, 2021  **Personality & Individual difference** | 350 | Auth | Psychopthy |  | Well being |  | Authenticity and well-being depends on the personality trait |
| Womick et al., 2019  **Personality & Individual difference** | 468 | DT4 |  | Authenticity | Wellbeing | Self-determination theory | The Dark Tetrad exhibited higher levels of well-being when authenticity levels were low. |
| Limone et al., 2020  **Behavioral Sciences** | 275 | DT |  | Orientation to happiness | Subjective well being | Instrumental Theory | Dark traits typically do not prioritize seeking happiness and subjective well-being (SWB), contrasting with the brighter aspects of narcissism, which emphasize pursuing emotional well-being through engaging activities. |
| Torres-Marín et al., 2022  **Personality & Individual difference** | 336 | DT |  |  | Humor |  | Narcissism predicted positive humor and enjoyment in being laughed at. Psychopathy correlated with aggressive humor and sarcasm. Machiavellianism was linked to ironic-satirical humor. Sadism related to darker humor dimensions. |
| Womick et al,2020  **Personality & Individual difference** | 512 | N |  | Subjective well being  Self-esteem | Meaning in Life (MIL) | Attachment theory | Narcissism enhances subjective well-being (SWB) through MIL and the routes from narcissism to SWB via MIL and self-esteem operate independently. |
| Yang et al, 2022  **Frontier in Psychology** | 620 | M |  | gratitude | Learning oriented subjective well-being | Life-history theory  Construction Theory | Gratitude partially mediates the relationship between Machiavellianism and learning oriented subjective well-being |
| García et al,2024  **Personality & Individual difference** | 390 | DT |  | Self-esteem | Humor Styles |  | Self-esteem and self-defeating contribute significantly to explore the dark triad humor style |
| Gómez‐Leal et al, 2023  **Scandinavian Journal of Psychology** | 345 | DT |  | cognitive-emotional regulation strategies | Affective and subjective well-being |  | Individuals exhibiting high levels of the Dark Triad traits demonstrated a distinct emotion regulation style affecting their subjective well-being (SWB). |
| Jonason et al,2015b  **Psychology of Aesthetics, Creativity, and the Arts** | 280 | DT |  | Gender | Creativity |  | Narcissism predictive of high creativity,psychopaths predicts creativity in mechanical task as compared to task with scholarly skills,Machiavellianism has shown little variance for creativity |
| Papageorgiou et al,2017  **Personality & Individual difference** | 410 | N |  | Mental Toughness | Psychopathy |  | Narcissism demonstrates notable adverse indirect impacts on psychopathy mediated by MT |
| Kennedy et al,2021  **Frontier in Psychology** | 495 | DT |  | Affective Motivation to lead  Social Motivation to lead | Leadership intention |  | Dark triad leads to significant variance in motivation to lead and subsequently leadership intention |
| Stanwix & Walker,2021  **Personality & Individual difference** | 320 | DT |  |  | Risk taking | Reinforcement Sensitivity Theory | Except Machiavellianism all dark tetrad equally contributed to advantageous risk-taking |
| Renier & Toma, 2022  **Personality & Individual difference** | 365 | N | Situational factors (referent, dimension, and valence) |  | Transparent towards others (felt transparency) |  | A high narcissus feels transparent towards others. literature contributes to narcissism, metaperception, transparency. |
| Shah et al, 2022  **Personnel Review** | 290 | M | Transformational leadership  Transactional leadership |  | Citizenship behavior, counterproductive work behavior | Leadership theory | Transactional leadership contributed to moderate toward citizenship behavior and positive outcomes |
| Frazier et al,2020  **Journal of Leadership & Organizational Studies** | 242 | M |  | Employee shared perception of leadership  Employee psychological empowerment | Team member performance  Organizational performance | Social learning theory  Role theory | Contribute to explore how Machiavellinist leaders effects employee perception. |
| Zhu et al,2021  **Personality & Individual difference** | 488 | M | Life style  Age  Gender  Social support |  | Quality of life |  | Contributed by using lifestyle as a mechanism to explore the quality of life of Mach via large sample |
| Nedeljković et al,2023  **Personality & Individual difference** | 310 | DT |  | Anger rumination | Forgiveness | Integrative Cognitive Model of trait anger and reactive aggression | Anger rumination lowers the dark triad feelings for revenge and fully mediate the relationship with forgiveness |
| Lee & Gibbons,2017  **Personality & Individual difference** | 375 | DT |  | empathic and emotion processes (recovery, reactivity, empathy) | Trait Compassion  State compassion | callousness theory of DT | Psychopathy and narcissism predicted state compassion. Overall DT lack correlated to compassion |
| Kaja et al, 2021  **Personality & Individual difference** | 290 | DT |  | Cognitive emotion regulation strategies | Emotional states |  | Maladaptive cognitive emotion regulation strategies mediate the link between narcissism/Machiavellianism and stress, anxiety, and depression. Conversely, adaptive strategies mediate the connection between psychopathy and depression. |
| Zuo et al, 2016  **Personality & Individual difference** | 432 | N |  | Self-esteem | Personal Morality (symbolization, internalization, pro social behavior |  | Narcissism outperformed Machiavellianism and psychopathy in moral identity and prosocial behavior. Notably, its positive effects on symbolization and prosocial behavior were more pronounced with lower self-esteem compared to higher self-esteem. |
| Denovan et al,2021  **Learning and individual difference** | 540 | DT |  | Mental Toughness | Learning Style (depression, strategic learning, surface learning) |  | Narcissism indirectly contributed to deep learning, enhanced strategic learning, and reduced symptoms of depression. Psychopathy and Machiavellianism predicted surface learning. |
| Jungst et al,2021  **Personality & Individual difference** | 312 | M | Social sources, relationship quality |  | Work engagement | Conservation of Resource theory | The social sources and relationships effects were less pronounced at higher levels of machiavellianism |
| Belschak et al,2018  **Frontier in Psychology** | 385 | M |  |  | Affiliative OCB  Emotional manipulation  Knowledge hiding |  | Under conditions of high ethical leadership the negative tendencies of Machiavelli’s may reduce such as emotional manipulation and enhances Mach prosocial behavior |
| Khan et al,2020  **Personnel Review** | 310 | P | HR Practices | Self-efficacy | Career satisfaction  Turnover intention | Conservation of resource theory | psychopathic leadership lowers employee career satisfaction. Additionally, the Human Resource Practitioner's (HRP) supportive role diminishes turnover intentions by boosting self-efficacy |
| Du & Templer ,2021  **Journal of Happiness Studies** | 405 | P |  | Boldness | Subjective wellbeing  Job satisfaction  Life satisfaction  Positive effect  Negative effect | Self-determination theory  Personality Theory | Boldness serves as a protective factor in successful psychopathy and increase job satisfaction, whereas meanness diminishes job satisfaction |
| Djeriouat,2024  **Personality & Individual difference** | 360 | DT |  |  | Free will intuitions  Moral responsibility | philosophical intuitions regarding freedom and moral responsibility. | psychopathy and, to a lesser extent, narcissism were associated with lower agreement regarding agents' possession of freedom and moral responsibility. Machiavellianism did not show a significant link with these philosophical intuitions. |
| Wang et al, 2023  **Journal of Applied Psychology** | 580 | EL | Leader Narcissism | Self-efficacy  Social admiration | Leader effectiveness | Self-concordance theory | Leaders with narcissistic traits tend to exhibit more ethical behavior, albeit at a higher motivational cost and with fewer social benefits than their less narcissistic peers. |
| Schyns et al,2023  **Personality & Individual difference** | 340 | N |  | Identity (Individualist vs collectivist) | Motivation to lead | Relational identity theory | Narcissism emerges as an antecedent of motivation to lead with full mediation of individual cultural identity |
| Wang et al, 2024  **Organizational Behavior and Human Decision Processes** | 495 | N |  | Self-motive(rivalry/admiration)  Social motive (group identification) | Knowledge sharing | Trait activation theory | Positive effects of narcissism on knowledge sharing through self and social motives |
| Spurk & Hirschi, 2018  **European Journal of Work and Organizational Psychology** | 456 | DT | Organizational change  Age |  | psychological climate at work | selection–evocation–manipulation theory | Within the group of employees who transitioned between organizations, the Dark Triad traits exhibited a notably stronger positive correlation with the shift in competitive psychological climate |
| Gao et al,2023  **Psychology Research and Behavior Management** | 520 | N | Legal environemnt |  | Employee welfare  Charity | Behavioral finance theory and stakeholder theory | The legal framework will mitigate the impact of CEO narcissism on philanthropic contributions and employee well-being. |
| Wang et al,2021  **Basic and Applied Social Psychology** | 375 | N | Power distance orientation | Affective organizational committment | Employee performance | Social Exchange Theory | Power distance orientation moderates the impact. For those with high power distance, the link between supervisor narcissism and organizational commitment, as well as performance, weakens.  Top of Form |
| Hafeez et al., 2025 | 506 | P | AI Assistants | Self-efficacy | Employee performance | Social Cognitive Theory |  |

Note**.DT:** Dark Triad, **DT4:** Dark Tetrad, **N:** Narcissism,M: Machiavellianism, **P:** Psychopathy, **UB:** Unethical Behavior**, EL:**Ethical leadership, **EP:**Entrepreneurial Capability, **Auth:** Authenticity, [**IM:** Impression](IM:Impression) Management
